# Long-term high-protein diet intake accelerates adipocyte senescence through macrophage CD38-mediated NAD^+^ depletion

**DOI:** 10.1016/j.molmet.2025.102306

**Published:** 2025-12-13

**Authors:** Xiaohan Yang, Lun Hua, Dengfeng Gao, Yanni Wu, Yi Yang, Xianyang Jin, Xuemei Jiang, Chao Jin, Bin Feng, Lianqiang Che, Shengyu Xu, Yan Lin, Long Jin, Yong Zhuo, Mingzhou Li, De Wu

**Affiliations:** 1 Animal Nutrition Institute, Sichuan Agricultural University, Chengdu, 611130, China; 2 State Key Laboratory of Swine and Poultry Breeding Industry, College of Animal Science and Technology, Sichuan Agricultural University, Chengdu, 611130, China; 3 Key Laboratory for Animal Disease-Resistant Nutrition of the Ministry of Education of China, Sichuan Agricultural University, Chengdu, 611130, China; 4 Animal Nutrition and Efficient Feed Utilization Key Laboratory of Sichuan Province, Sichuan Agricultural University, Chengdu, Sichuan 611130, China

**Keywords:** High protein diet, Nicotinamide adenine dinucleotide, Adipocyte senescence, CD38

## Abstract

High-protein (HP) diets are widely adopted in Western societies for body-weight management; yet, they exacerbate senescence-associated metabolic deterioration, posing an unresolved pathophysiological conundrum. Here, we demonstrate that long-term HP intake mediates adipocyte-specific NAD^+^ depletion and mitochondrial dysfunction in white adipose tissue (WAT). Single-nucleus transcriptomic analyses revealed adipocyte-restricted senescence signatures in HP-fed mice. Mechanistically, HP intake triggers macrophage-specific upregulation of CD38 (a key NAD^+^ hydrolase), which depletes adipocyte NAD^+^ pools and thereby accelerates cellular senescence. Restoration of NAD^+^ levels, either via supplementation with NAD^+^ precursor or pharmacological inhibition of CD38 activity, alleviated the senescence-associated metabolic sequelae induced by HP diets. Our findings establish macrophage-adipocyte NAD^+^ crosstalk as a central axis linking dietary protein excess to WAT aging, providing actionable targets for the prevention and treatment of age-related metabolic disorders.

## Highlights

• Long-term HP intake mediates adipocyte-specific NAD^+^ depletion and mitochondrial dysfunction in WAT.
• Single-nucleus transcriptomic reveals adipocyte-restricted senescence signatures in HP-fed mice.
• HP intake triggers macrophage-specific upregulation of CD38, which depletes adipocyte NAD^+^ pools and thereby accelerates cellular senescence.
• Restoring NAD^+^ levels, via NAD^+^ precursor supplementation or pharmacological inhibition of CD38 activity, mitigates HP diet-induced senescence-associated metabolic sequelae.

## Graphical abstract

**Figure:**
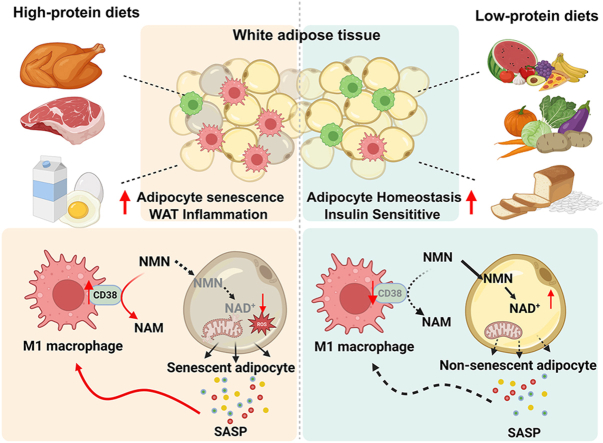

## 1 Introduction

Obesity drives an escalating global health crisis through insulin resistance, multi-organ dysfunction, and accelerated aging [1,2]. Short-term high-protein (HP) interventions contribute to body-weight management by augmenting satiety and stimulating thermogenesis [[3], [4], [5], [6]]. For humans, the Recommended Daily Intake (RDI) of protein to maintain nitrogen balance is 0.8 g·kg^−1^·d^−1^, representing ∼11% of total daily energy intake [7]. Higher protein intake is generally recommended for older adults to counteract age-related declines in muscle and bone protein synthesis, while children and adolescents also require increased protein intake due to their elevated anabolic demands [8,9]. In Western societies, average protein intake exceeds the RDI, with roughly one-quarter of the population consuming more than twice the recommended amount, i.e., over 1.6 g·kg^−1^·d^−1^ or 22% of daily energy derived from protein [[10], [11], [12]]. In contrast, both epidemiological and experimental evidence consistently associate moderate protein restriction (PR) with reduced age-related morbidity and prolonged lifespan across species [[13], [14], [15], [16]]. This paradox underscores an urgent necessity to clarify how dietary protein intake modulates organ-specific aging trajectories, particularly within metabolically dynamic microenvironments such as adipose tissue.

White adipose tissue (WAT) dysfunction constitutes a critical pathological nexus that links obesity to systemic aging [17,18]. Under metabolic stress, senescent adipocytes accumulate and secrete pro-inflammatory adipokines (most notably, IL-6 and TNFα) through the senescence-associated secretory phenotype (SASP) [[19], [20], [21]]. This pathological process sustains chronic low-grade inflammation and drives the propagation of secondary senescence across anatomically distant organs [18,22]. Although HP diets have been demonstrated to confer anti-obesogenic benefits in short-term studies [4,23], their long-term impacts on WAT senescence remain inadequately characterized. Recent observations further suggest a paradoxical association between HP intake and accelerated hepatic senescence [24], which underscores the organ-specific complexity of protein metabolism in the context of aging and presents a significant mechanistic conundrum.

Nicotinamide adenine dinucleotide (NAD^+^), a central redox cofactor and signaling metabolite, has emerged as a key regulator of the aging process [25,26]. The metabolic balance of NAD^+^ is closely associated with metabolic dysfunction induced by obesity [27,28]. Consistently, reduced NAD^+^ levels have been observed in the adipose tissues of obese rodents and human subjects [27,29,30]. Restoration of NAD^+^ through supplementation with its precursors has been shown to alleviate obesity-induced functional deterioration [27,28,31]. Cellular NAD^+^ homeostasis is maintained through the equilibrium between biosynthetic and degradative enzymes [32,33]. Among these enzymes, CD38, an ecto-NADase predominantly expressed in immune cells and endothelial cells, plays a critical role in driving WAT inflammation and amplifying SASP signaling [[34], [35], [36]]. However, the impact of dietary macronutrient composition, particularly protein, on the NAD^+^-CD38 axis remains elusive, which impedes its translational application in therapeutic settings.

Here, we investigate whether long-term HP intake accelerates WAT senescence through CD38-mediated NAD^+^ dysregulation. Utilizing multi-omics and genetic approaches, we demonstrate that long-term HP feeding induces adipocyte-specific NAD^+^ depletion and senescence by activating macrophage CD38. Pharmacological restoration of NAD^+^ or genetic ablation of macrophage-specific *Cd38* effectively attenuated HP-induced adipocyte senescence. These findings establish macrophage-adipocyte NAD^+^ crosstalk as an essential mechanism linking excessive dietary protein intake to WAT dysfunction, and identify potential therapeutic strategies to counteract obesity- and aging-related comorbidities.

## 2 Results

### 2.1 Long-term HP diets intake induces senescence in white adipose tissues

**Figure 1:**
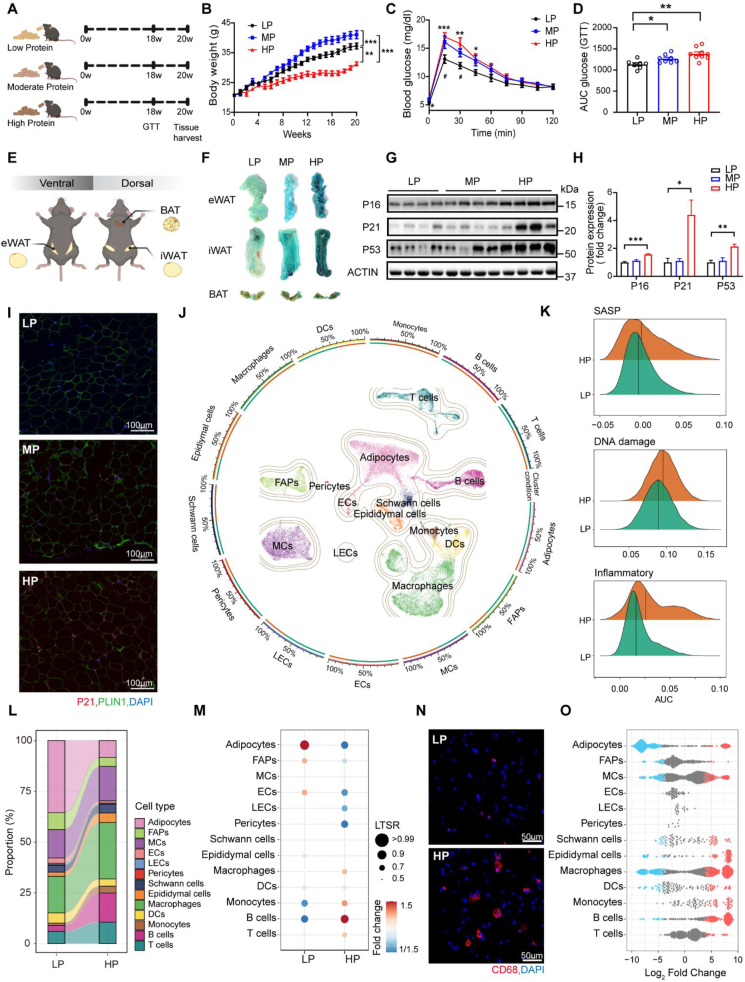
**Long-Term HP Diets Induce WAT-Specific Senescence.** (A) Experimental schematic. Male C57BL/6J mice were fed isocaloric diets with graded protein-carbohydrate ratios (LP: 9% protein, MP: 22% protein, and HP: 56% protein) for 20 weeks. (B) Body weight measured weekly for each mouse (*n* = 7). (C,D) Glucose tolerance test (GTT) performed after an overnight fast at week 18 (C) and corresponding calculation of the area under the curve (AUC) (D) (LP, *n* = 7; MP, *n* = 8; HP, *n* = 9). ∗*P* < 0.05, ∗∗*P* < 0.01, and ∗∗∗*P* < 0.001, LP vs HP; ^#^*P* < 0.05, LP vs MP. (E,F) Schematic of sample collection (E) and representative images of SA-β-gal staining in three representative adipose depots (i.e., epididymal white adipose tissue [eWAT], inguinal white adipose tissue [iWAT], and brown adipose tissue [BAT]) (F). (G,H) Western blot analysis of three senescence markers (P16, P21, and P53) in eWAT. Representative blots (G) and quantification results (H). (I) Representative immunofluorescence images of P21 (red), PLIN1 (green) and DAPI (blue) in eWAT. (J) Uniform manifold approximation and projection (UMAP) visualization of 34,907 profiled nuclei from eWAT. Colors indicate major cell-type clusters, which are defined by marker genes. (K) Ridge plot illustrating the comparison of activity scores for gene sets related to SASP, DNA damage, and inflammation. (L) Sankey diagram showing shifts in cellular composition between mice fed the HP diets and those fed the LP diets. (M) Relative proportional changes of each cell type in eWAT from mice fed the HP diets versus the LP diets. The color scale represents the fold change, and the dot size shows the probability of change (local true sign rate [LTSR]), which was calculated using a generalized linear mixed model with a Poisson distribution outcome. (N) Representative immunofluorescence images of CD68 (red) with DAPI (blue) staining in eWAT. (O) Bee-swarm plot showing the relative proportional changes for each cell type using Milo algorithm. Neighborhoods with spatial false discovery rate (FDR) ≤ 10% are highlighted. Data are presented as mean ± s.e.m. Unpaired Student's *t*-test was used for statistical analysis. ∗*P* < 0.05, ∗∗*P* < 0.01, and ∗∗∗*P* < 0.001. Each symbol represents one biological replicate.

To evaluate the metabolic consequences associated with long-term intake of a high-protein (HP) diet, we subjected 8-week-old male C57BL/6J mice to an HP feeding regimen (56% protein content) for a 20-week duration. For comparison, two additional groups of mice were separately maintained on isocaloric diets with protein-to-carbohydrate ratios of 22% (moderate protein, MP) and 9% (low protein, LP), respectively (Figure 1A). Compared with mice fed the MP or LP diet, those administered the HP diet demonstrated a reduction in both food intake and fat mass (Supplemental Fig. S1A–C); however, this lean phenotype of mice fed an HP diet was not accompanied by the achievement of systemic metabolic benefits, as evidenced by impaired (or non-improved) glucose tolerance (Figure 1B–D, and Supplemental Fig. S1D and E). Given the established link between adipose senescence and metabolic dysfunction [17,18], we next assessed cellular senescence via senescence-associated β-galactosidase (SA-β-gal; a well-recognized hallmark of cellular aging) staining (Figure 1E,F). HP-fed mice displayed robust SA-β-gal activity in white adipose tissue (WAT), particularly in the epididymal WAT (eWAT) and inguinal WAT (iWAT) depots (Figure 1F). Consistently, canonical senescence markers were upregulated at both the transcript and protein levels in eWAT and iWAT of HP-fed mice (Figure 1G–I, and Supplemental Fig. S1F–I). By contrast, brown adipose tissue (BAT) showed no detectable senescence signatures (Figure 1F, and Supplemental Fig. S1J-L). These findings suggest that long-term HP intake acts as a senescence inducer specifically in WAT.

Considering the high cellular heterogeneity of WAT and the pronounced inflammatory and aging features of eWAT [37], we next sought to delineate the cell type-specific alterations induced by HP diet feeding within this depot. Consistent with previous studies [[13], [14], [15], [16]], we observed that long-term intake of an LP diet confers more pronounced metabolic benefits (Figure 1C,D, and Supplemental Fig. S1D and E). To clarify the WAT aging trajectories induced by HP-diet intake, we designated the LP group as the control group for subsequent comparative analyses. Analysis of 34,907 nuclei using Uniform Manifold Approximation and Projection (UMAP) revealed 13 major cell types, which were defined based on the expression of canonical marker genes (Figure 1J, and Supplemental Fig. S2A and B). Consistent with the accumulation of senescent cells (Figure 1F–I), the eWAT of mice fed a HP-diet exhibited enrichment of genes related to processes of SASP, DNA damage, and inflammation (Figure 1K). Compared with the control mice, the HP-fed mice exhibited a marked reduction in adipocyte population, with the proportion of total nuclei decreasing from 35.5% in control mice to 8.4% in HP-fed mice. This reduction was accompanied by an expansion of immune cells, particularly macrophages, whose proportion increased from 18.0% in control mice to 27.8% in HP-fed mice (Figure 1L,M). This expansion was further corroborated by immunofluorescent staining of CD68 (a macrophage marker) (Figure 1N). These profound cellular alterations were recapitulated by Milo differential abundance analysis [38] (Figure 1O). This inverse pattern in cell population between adipocytes and macrophages suggests a pathognomonic feature of HP-fed WAT.

### 2.2 HP diet specifically induces adipocyte senescence

**Figure 2:**
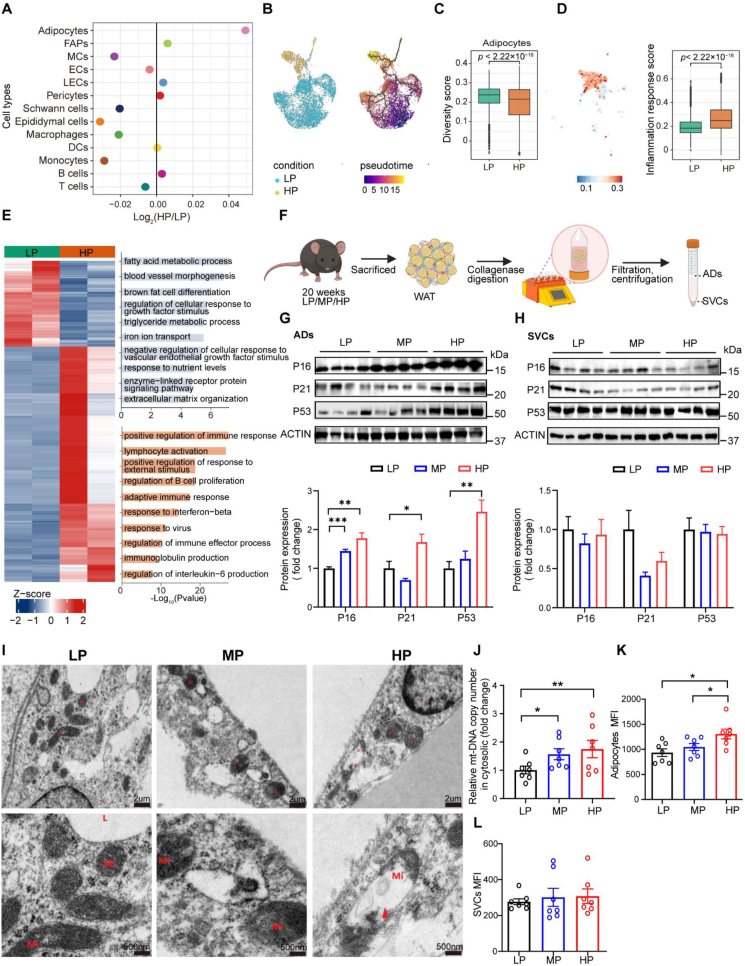
**Chronic HP Diets Drive Adipocyte-Specific Senescence**. (A) Dot plot showing the log_2_ ratio of transcriptional heterogeneity among individual nuclei (HP/LP) for cell types in eWAT. (B) Monocle3 pseudotemporal ordering of adipocyte nuclei. UMAP/trajectory plots are colored by condition (HP vs LP) (B:left) and by inferred pseudotime (B:right). (C) Diversity scores calculated using cell-type-specific HVG gene sets derived of LP group. (D) Inflammation response score calculated based on the gene set annotated to the inflammation response pathway. (See Methods for details.) (E) Heatmap of differentially expressed genes with pathway annotations in adipocytes. (F–H) Experimental schematic of sample collection (F) and Western blot analysis of senescence markers (P16, P21and P53) in adipocytes and SVCs isolated from eWAT (G,H). (I)Transmission electron microscopy image and the representative images of impaired mitochondria. (J) Related cytosolic mitochondrial DNA levels (*n* = 7). (K,L) Reactive oxygen species in adipocytes (J) and SVCs (K) isolated from eWAT of mice fed a HP diets (*n* = 7). Data are presented as mean ± s.e.m. Unpaired Student's *t*-test was used. ∗*P* < 0.05, ∗∗*P* < 0.01, and ∗∗∗*P* < 0.001. Each symbol represents one biological replicate.

An emerging characteristic of cellular senescence is the heightened transcriptional heterogeneity observed among individual cells or nuclei within specific cell types, rather than being a consequence of a coordinated transcriptional program [39]. Among the 13 distinct cell types examined, adipocytes from HP-fed mice exhibited the most pronounced increase in transcriptional variability (Figure 2A), accompanied by a marked reduction in differentiation potential (Figure 2B, and Supplemental Fig. S3A). Loss of cell identity constitutes another hallmark of cellular senescence [40,41]. Consistent with this notion, adipocytes in HP-fed mice showed impaired maintenance of cell identity (Figure 2C, and Supplemental Fig. S3B). The inflammation response score was significantly higher in adipocytes from HP-diet (Figure 2D). Differential expression analysis further revealed that genes downregulated in adipocytes from HP-diet mice were enriched in metabolic processes (e.g., fatty acid metabolism, and nutrient response), whereas upregulated genes were enriched in immune-related pathways (e.g., immune response regulation, and immunoglobulin production) (Figure 2E). Consistent with these transcriptomic signatures, the protein levels of senescence markers (P16, P21, and P53) were highly selectively expressed in adipocytes, but not in stromal vascular cells (SVCs) from HP-fed mice (Figure 2F–H), highlighting the adipocyte-specific vulnerability to senescence induction. These results indicate that long-term HP-diet intake preferentially induces senescence in adipocytes.

Mitochondrial dysfunction is a canonical hallmark of cellular senescence [[42], [43], [44]]. Transmission electron microscopy revealed abnormal mitochondrial morphology in adipocytes from HP-fed mice (Figure 2I), accompanied by elevated cytosolic mitochondrial DNA (mt-DNA) (Figure 2J), indicating that long-term HP diet intake induces mitochondrial stress in adipocytes. Mitochondrial reactive oxygen species (ROS) accumulation impairs mitochondrial function while simultaneously acting as signaling molecules to trigger cellular senescence [45,46]. Thus, we hypothesized that mitochondrial ROS accumulation might potentiate cellular senescence in adipocytes of HP-fed mice. To address this, we quantified the ROS levels in adipocytes and SVCs isolated from eWAT by flow cytometry. Indeed, ROS accumulation was significantly elevated in adipocytes but not in SVCs of HP-fed mice (Figure 2K,L), implicating mitochondrial ROS accumulation as a key driver of adipocyte senescence under long-term HP feeding.

Collectively, these data indicate that prolonged HP feeding promotes adipocyte mitochondrial dysfunction and ROS accumulation which likely contribute to adipocyte senescence.

### 2.3 HP diet induces NAD^+^ depletion in adipocytes

**Figure 3:**
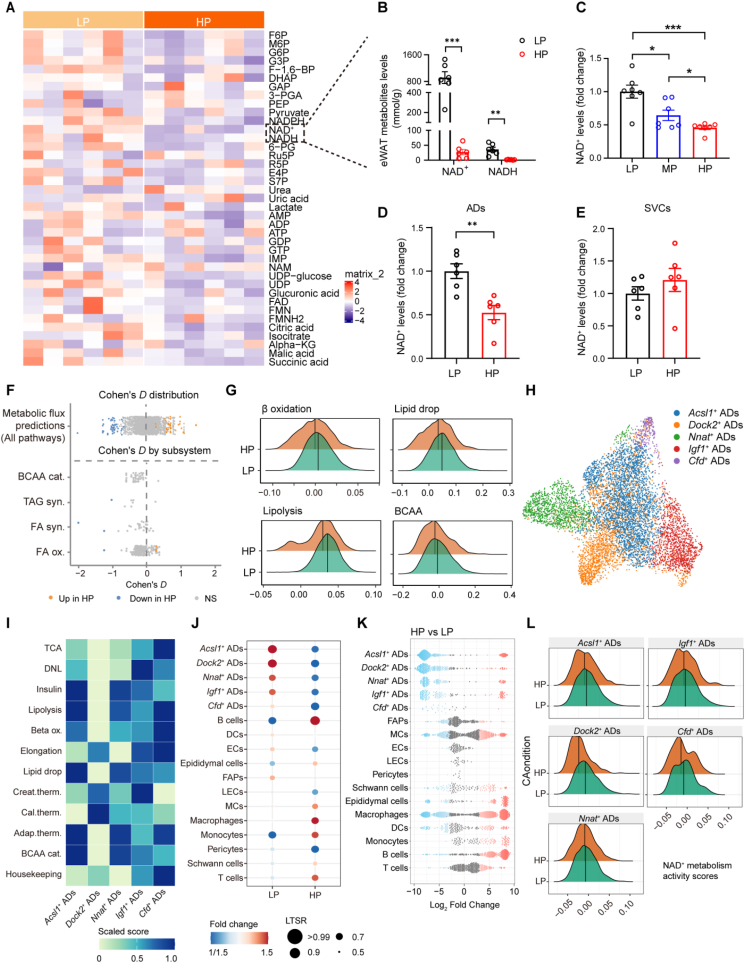
**Long-Term HP Intake Drives Adipocyte NAD^+^ Depletion**. (A) Heatmap showing differentially abundant metabolites in eWAT from mice fed an HP diets versus an LP diets. (B,C) Differential metabolites involved in NAD^+^ metabolism, as measured by mass spectrometry (B) and confirmed by enzymatic cycling assay (C) (*n* = 7). (D,E) NAD^+^ levels measured in adipocytes (D) and SVCs (E) isolated from eWAT of mice fed an HP or LP-fed diets (*n* = 6). (F) Transcriptomic-informed flux analyses of metabolic reactions in adipocytes from eWAT of mice fed a HP or LP-fed diets. Global (upper) and representative (lower) metabolic pathways. Reaction level and Cohen's *D* values are colored by p value < 0.05 (Wilcoxon): red, higher in HP mice; blue, lower in HP mice; grey, non-significant. (G) Pathway activity scores for major metabolic pathways in individual adipocytes. (H) UMAP plot of adipocytes pooled across conditions. (I) Overall activity in metabolic pathways in adipocyte subpopulations (scaled mean scores). Adap., adaptive; Therm., thermogenesis; DNL., *de novo* lipogenesis; ox., oxidation; TCA., tricarboxylic acid cycle; Cal., calcium; Creat., creatine; cat.,catabolism. (J,K) Adipocyte subpopulations proportions in the combined cohort analyzed by LTSR (J) and Milo algorithms (K). (L) Ridge plots comparing NAD^+^ metabolism activity scores across adipocyte subpopulations. Data are presented as mean ± s.e.m. ∗*P* < 0.05, ∗∗*P* < 0.01, and ∗∗∗*P* < 0.001. Each symbol represents one biological replicate.

Mitochondrial impairment and metabolites alterations are tightly interconnected, with dysfunctional mitochondria disrupting cellular metabolism, while aberrant metabolite profiles further aggravate mitochondrial dysfunction [47,48]. To identify metabolites associated with HP diet-induced mitochondrial impairment, we performed targeted metabolomic analyses, focusing primarily on tricarboxylic acid cycle (TCA), glycolysis, and pentose phosphate pathway in the eWAT of mice fed either an HP or LP diet. Given the essential role of NAD^+^ in mitochondrial function [49,50], we examined NAD^+^ homeostasis and found a significant depletion of both NAD^+^ and NADH in eWAT of HP-fed mice (Figure 3A,B). This finding was further confirmed by enzymatic cycling assays (Figure 3C). To further characterize cell type-specific alterations in NAD^+^ metabolism associated with HP-diet intake, we fractionated eWAT into adipocytes and SVCs. Intriguingly, we found that adipocytes from HP-fed mice exhibited more pronounced losses of NAD^+^ compared to SVCs (Figure 3D,E), highlighting adipocyte-specific vulnerability to HP dietary intervention.

To further characterize adipocyte metabolic reprogramming, we applied compass-based metabolic flux modeling to transcriptomic data [51]. This analysis revealed a unique activation state specific to adipocytes of eWAT from HP diet-fed mice, encompassing both known and previously uncharacterized metabolic perturbations (87 out of 1,895 reactions) (Figure 3F). Specifically, compared to adipocytes from LP-fed mice, those from HP diet-fed mice displayed significant defects in metabolism of fatty acids and branched-chain amino acids (BCAA) breakdown (Figure 3F), providing further evidence of impaired metabolic flexibility under HP dietary conditions. Assessments based on enzymatic activity scores and analysis of pathway-limiting enzymes consistently demonstrated reductions in key metabolic activities in adipocytes from HP diet-fed mice (Figure 3G).

We next aimed to investigate the molecular characteristics and diverse metabolic functions underlying the profound phenotypic changes in adipocytes from HP-fed mice. Fine cluster annotation performed based on key marker genes identified five sub-populations within the adipocytes (Figure 3H, and Supplemental Fig. S3C), all of which exhibited reduced metabolic turnover (Figure 3I). HP feeding resulted in marked reductions in cell numbers across all adipocyte subpopulations (Figure 3J,K), suggesting increased cell death and/or a failure to replenish adipocytes. Given the functional coupling of NAD^+^ metabolism with metabolic flexibility, we systematically evaluated NAD^+^ metabolic dynamics using single-cell gene-set scoring. Notably, *Dock2*^*+*^ and C*fd*^*+*^ adipocyte subpopulations showed the most prominent reductions in NAD^+^ metabolic activity (Figure 3L). This spatially restricted metabolic reprogramming coincided with the onset of cellular senescence, implicating NAD^+^ depletion as a mechanistic link between HP feeding and adipocyte senescence.

### 2.4 HP diet enhances activity of NADase CD38 in macrophages

**Figure 4:**
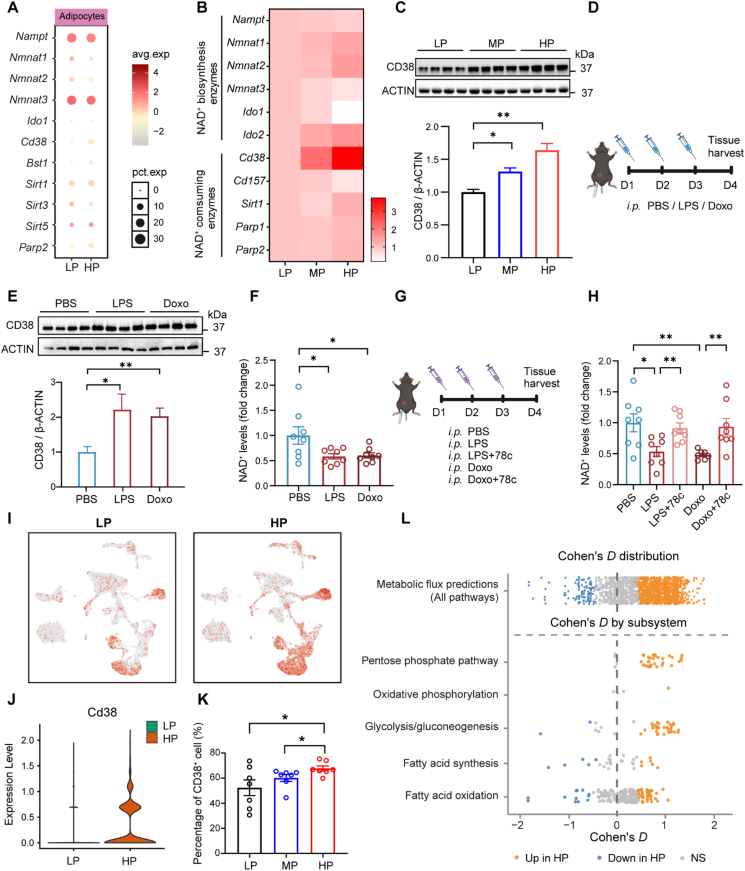
**Long-term HP Intake Increases NADase CD38 Levels in Macrophages**. (A) mRNA expression of NAD^+^ metabolic enzymes in adipocytes analyzed by snRNA-seq. (B) mRNA expression of NAD^+^ metabolic enzymes in eWAT from mice fed LP, MP, or HP diets (LP, *n* = 8; MP, *n* = 7; HP, *n* = 9). (C) Western blot analysis of CD38 protein levels in eWAT. (D,E) Schematic diagram of inflammatory (LPS) or senescence (doxo) induction in mice (D) and Western blot analysis of CD38 protein levels in eWAT (E). (F) Levels of NAD^+^ in eWAT following treatment with LPS or Doxo (*n* = 8). (G,H) Schematic diagram of CD38 inhibition (78c) following LPS or doxo induction (G) and NAD^+^ levels in eWAT after 78c treatment (H) (*n* = 7–8). (I,J) UMAP plots showing *Cd38* expression across samples in eWAT (I) and violin plot comparing *Cd38* expression in macrophages (J). (K) Quantification of CD38^+^F4/80^+^ macrophages in eWAT from HP- or LP-fed mice, measured by FACS (*n* = 7). (L) Transcriptomic flux analyses of global (upper) and representative (lower) metabolic pathways in macrophages from HP-fed diets versus with LP-fed diets. Reaction level and Cohen's *D*, coloured by FDR <0.05 (Wilcoxon): red, HP high; blue, HP low; grey, non-significant. Data are presented as mean ± s.e.m. Unpaired Student's *t*-test was used. ∗*P* < 0.05, ∗∗*P* < 0.01, and ∗∗∗*P* < 0.001. Each symbol represents one biological replicate.

To investigate the mechanism underlying NAD^+^ depletion in adipocytes, we examined the expression profiles of NAD^+^ biosynthetic and catabolic enzymes in adipocytes using snRNA-seq data. However, the expression levels of these enzymes were largely comparable between adipocytes derived from HP- and LP-fed mice (Figure 4A), suggesting an extrinsic cause of NAD^+^ depletion in adipocytes. In contrast, qPCR revealed a marked upregulation of the NAD^+^ hydrolase CD38 in eWAT from HP-fed mice, which was confirmed at the protein level by Western blotting (Figure 4B,C). These data implicate that CD38 is most likely involved in HP-induced NAD^+^ depletion in adipocytes.

We next investigated whether HP-associated stressors are capable of inducing CD38 expression. Administration of lipopolysaccharide (LPS, a well-characterized proinflammatory stimulus) or doxorubicin (doxo, a DNA-damaging agent), resulted in elevated CD38 protein levels and reduced NAD^+^ levels in WAT (Figure 4D–F). This set of observations recapitulated the phenotypic characteristics observed following long-term HP feeding. To further confirm the functional relevance of CD38 activity, we treated mice with 78c, a potent CD38 inhibitor, subsequent to LPS or doxo challenge (Figure 4G). Treatment with 78c effectively prevented the decline in NAD^+^ levels following LPS or doxo challenge (Figure 4H), indicating that early-onset inflammation and SASP activity drive CD38-dependent NAD^+^ depletion in WAT.

Considering that CD38 is predominantly expressed in immune cells and endothelial cells [52,53], we analyzed the snRNA-seq datasets to identify the cell populations responsible for CD38-mediated NAD^+^ depletion. Macrophages showed the highest *Cd38* expression, which was significantly elevated in HP-fed mice compared to LP-fed controls (Figure 4I,J). Flow cytometry confirmed an increased proportion of CD38^+^ cells within M1 macrophages under HP feeding (Figure 4K), indicating a unique activation state of these cells. Indeed, metabolic profiling further demonstrated that these macrophages underwent dual metabolic reprogramming, characterized by enhanced glycolysis (a pro-inflammatory feature) and elevated oxidative metabolism (an anti-inflammatory feature), together with shifts in the pentose phosphate pathway, TCA cycle, and lipid/cholesterol metabolism (Figure 4L). Collectively, these findings suggest that macrophage-derived CD38 activation mediates adipocyte NAD^+^ depletion in response to HP diets.

### 2.5 Macrophage-derived CD38 mediates adipocyte senescence induced by a HP diet

**Figure 5:**
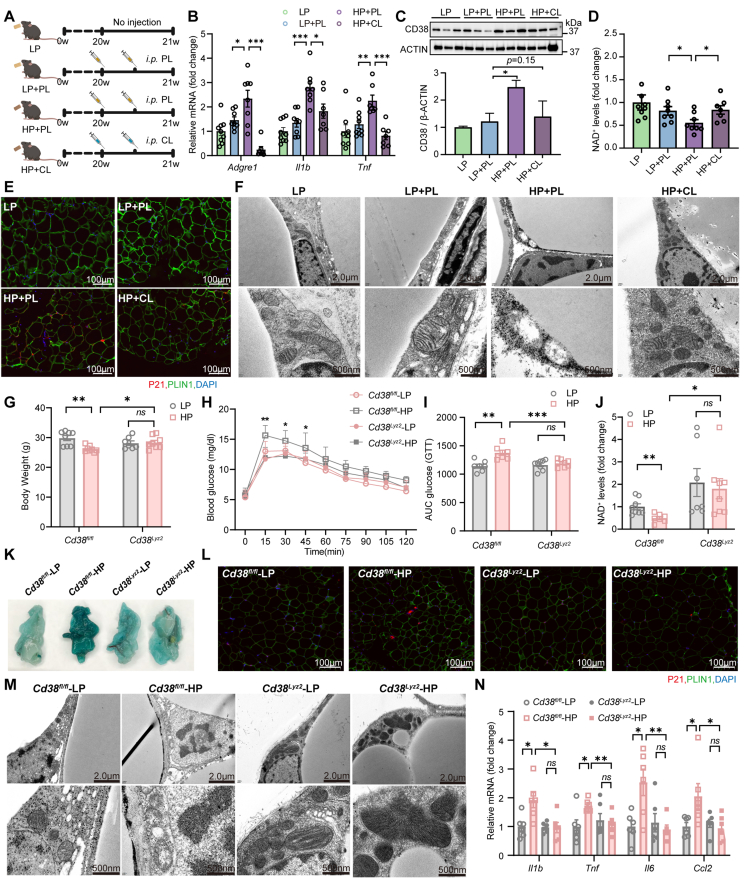
**Macrophage CD38 Mediates Adipocyte Senescence Induced by Long-Term HP Diet Intake**. (A) Schematic illustration of the macrophage depletion protocol: mice subjected to long-term HP-diet feeding were administered clodronate (CL) or control liposomes (PL) via intraperitoneal injection (200 μL per injection) twice weekly. (B) mRNA levels of *Adgre1* (a macrophage-specific marker) and two inflammatory cytokines (*Il1b* and *Tnf*) in eWAT of mice treated with PL or CL (*n* = 7–9). (C) Western blot analysis of CD38 protein in eWAT following macrophage depletion. (D) NAD^+^ concentrations in eWAT measured by mass spectrometry in CL- and PL-treated mice (*n* = 7–8). (E) Representative immunofluorescence staining images of eWAT from CL- and PL-treated mice (P21, red; PLIN1, green; DAPI, blue). (F) Representative TEM images of adipocytes isolated from CL- and PL-treated mice. (G) Body weight of WT or macrophage-specific *Cd38* ablation (*Cd38*^*lyz2*^) mice after 24 weeks of feeding with a standard diet or HP diet (*n* = 7–8). (H,I) Glucose tolerance test (G) and calculation of area under curve (H) were performed after overnight fast (*n* = 7–10). (J) NAD^+^ levels in eWAT (*n* = 7–8). (K) Representative SA-β-gal staining images of eWAT. (L) Representative images immunofluorescence staining of eWAT (P21, red; PLIN1, green; DAPI, blue). (M) Representative TEM images of eWAT and magnified views of impaired mitochondria. (N) mRNA levels of inflammatory genes (*Il1b, Tnf*, *Il6* and *Ccl2*) in eWAT (*n* = 6). Data are presented as mean ± s.e.m. ∗*P* < 0.05, ∗∗*P* < 0.01, and ∗∗∗*P* < 0.001. Each symbol represents one biological replicate.

To test whether macrophages drive CD38-mediated NAD^+^ depletion, we selectively depleted macrophages in HP-fed mice via intraperitoneal injection of clodronate liposomes (CL), with PBS liposomes (PL) serving as a control (Figure 5A). CL treatment effectively reduced the abundance of macrophages in WAT, as evidenced by decreased levels of *Adgre1* and pro-inflammatory cytokines (*Il1b* and *Tnf*) (Figure 5B). Notably, CD38 protein expression in WAT was markedly downregulated in CL-treated mice (Figure 5C), and the HP-induced reduction in NAD^+^ levels was significantly rescued (Figure 5D). In parallel, CL treatment attenuated the HP-induced upregulation of the senescence marker P21 and ameliorated mitochondrial damage (Figure 5E,F). This highlights the importance of macrophage-derived CD38 in WAT NAD^+^ loss under long-term HP intake.

To further validate the critical role of macrophage CD38 in HP-induced adipocytes senescence, we subjected mice with macrophage-specific *Cd38* ablation (*Cd38*^*Lyz2*^) to long-term HP feeding. Despite comparable body weight between HP- and LP-fed in *Cd38*^*Lyz2*^ mice (Figure 5G), macrophage CD38 deficiency alleviated HP-induced glucose intolerance (Figure 5H,I), suggesting that macrophage CD38 is critical to systemic metabolic dysregulation. Consistent with our previous findings that long-term HP intake reduces NAD^+^ levels in WAT (Figure 3A–C), NAD^+^ levels in WAT were comparable between LP- and HP-fed macrophage-specific *Cd38* knockout mice (*Cd38*^*Lyz2*^) (Figure 5J). This provides further evidence that macrophage CD38 is essential for the HP-induced decline in NAD^+^. WT mice fed a HP diet exhibited pronounced senescence in WAT, whereas *Cd38*^*Lyz2*^ mice showed attenuated senescence specifically in eWAT (Figure 5K,L). Concomitantly, the HP-induced upregulation of senescence markers, mitochondrial defects and transcript levels of proinflammatory cytokines were mitigated by macrophage CD38 ablation (Figure 5K-N), confirming its necessity in diet-accelerated adipose senescence. Together, these findings demonstrate that macrophage-derived CD38 is essential for HP diet-induced NAD^+^ depletion, adipocyte senescence, and metabolic dysfunction.

### 2.6 Supplementation with NAD ^+^ precursor or inhibition of CD38 activity alleviates HP diet-induced WAT senescence

**Figure 6:**
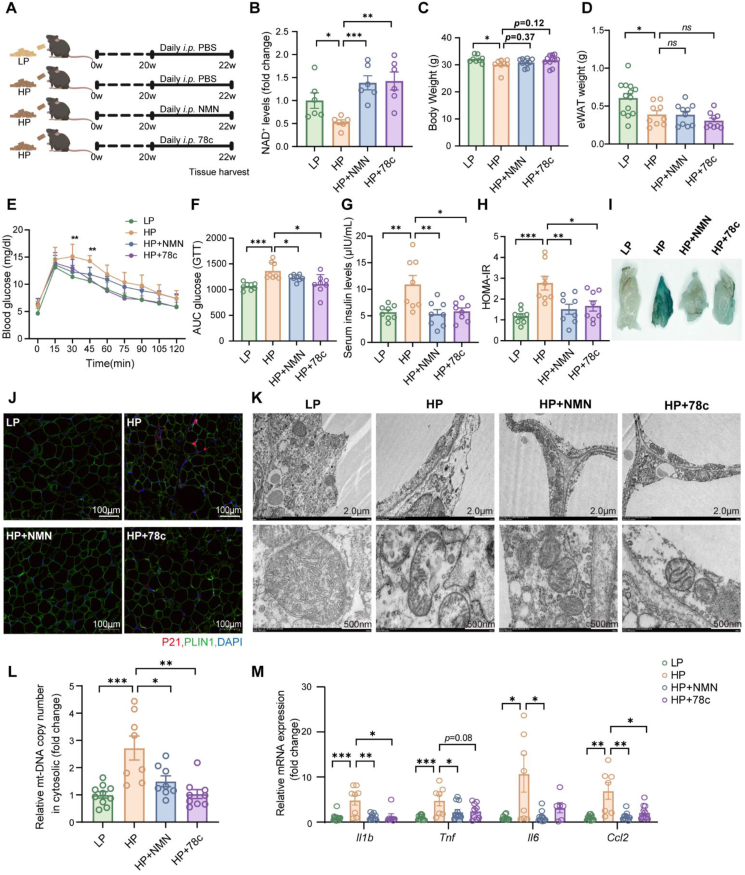
**Supplementation with NAD^+^ Precursor or Inhibition of CD38 Activity Alleviates HP Diet-Induced Senescence in WAT**. (A) Schematic diagram of the experiment. After 20 weeks HP diet intake, mice received daily injections of NMN (400 mg/kg) or 78c (20 mg/kg) for two weeks. (B) NAD^+^ levels in eWAT (*n* = 6). (C) Final body weight (*n* = 8–11). (D) Weight of eWAT (*n* = 8–13). (E,F) Glucose tolerance test (E) and calculation of area under curve (F) were performed after an overnight fast (*n* = 8). (G,H) Concentration of serum insulin (G) and insulin resistance index (H) (*n* = 8). (I) Representative SA-β-gal staining images of eWAT. (J) Representative immunofluorescence staining images of eWAT (P21, red; PLIN1, green; DAPI, blue). (K) TEM images and the representative images of impaired mitochondria. (L) Relative levels of cytosolic mitochondrial DNA in adipocytes isolated from eWAT (*n* = 8–10). (M) mRNA levels of inflammatory marker genes (*Il1b, Tnf*, *Il6* and *Ccl2*) in eWAT (*n* = 6–12). Data are presented as mean ± s.e.m. ∗*P* < 0.05, ∗∗*P* < 0.01, and ∗∗∗*P* < 0.001. Each symbol represents one biological replicate.

To test the therapeutic efficacy of NAD^+^ repletion in the context of long-term HP feeding, we administered nicotinamide mononucleotide (NMN, an NAD^+^ precursor) or the CD38 inhibitor (78c) on a daily basis for two weeks subsequent to 20 weeks of HP-diet intake (Figure 6A). As expected, both interventions effectively restored NAD^+^ levels in WAT (Figure 6B), without inducing alterations in body weight or adiposity (Figure 6C,D). Notably, treatment with NMN and 78c resulted in a significant improvement in glucose tolerance (Figure 6E–H). Subsequent characterization assays revealed that WAT senescence signatures, quantified by β-galactosidase activity and protein levels of P21 by immunofluorescence, were suppressed in both treatment groups (Figure 6I,J). These results indicate that restoration of NAD^+^ homeostasis in WAT attenuates adipocyte senescence and improves insulin sensitivity in the setting of long-term HP intake.

Next, we evaluated the impact of NAD^+^ levels repletion on HP-induced mitochondrial damage. The number of abnormally structured mitochondria was markedly reduced in mice treated with NMN or 78c (Figure 6K), a finding further supported by the mitigation of mitochondrial DNA amounts in adipocyte cytosol (Figure 6L). To characterize the effects of restoring NAD^+^ homeostasis on WAT inflammation, we measured the expression of four key inflammatory marker genes. We observed that WAT from HP-fed mice exhibited elevated expression of these inflammatory markers; however, their expression was restored to baseline levels in mice treated with NMN or 78c (Figure 6M). This finding further establishes NAD^+^ repletion as a critical checkpoint for preventing WAT dysfunction.

Together, these findings establish NAD^+^ depletion as a central node linking long-term HP feeding to adipocyte senescence, and show that either precursor supplementation or CD38 inhibition is sufficient to preserve WAT homeostasis and systemic metabolic health.

## 3 Discussion

HP-diets are widely advocated for body-weight management; however, they exacerbate senescence-associated metabolic decline, presenting a mechanistic conundrum [5,6,24]. In the present study, we identify tissue-resident macrophages as key regulators of adipocyte NAD^+^ metabolism under long-term HP diet intake. Specifically, macrophage-derived CD38 accelerates adipocyte senescence by depleting NAD^+^ pools, thereby establishing a mechanistic link between dietary protein excess and adipose tissue aging. By uncovering this macrophage-adipocyte crosstalk, our work reconciles epidemiological observations that associated high protein consumption with age-related morbidity.

Global protein intake has risen in parallel with the increasing prevalence of type 2 diabetes [54,55]. High protein intake is also advocated as a strategy to support weight management. Additionally, it has been proposed that middle-aged and older adults should consume at least 1.0–1.2 g·kg^−1^·d^−1^ of protein to prevent age-related loss of muscle mass [8]. Furthermore, protein intake levels of up to ∼35% of daily energy requirements are considered acceptable in human nutrition [7,10,56]. In this study, we observed that mice fed a long-term HP-diets (accounting for ∼56% of total daily energy intake) shown reduced insulin sensitivity and accelerated adipocyte senescence. It is noteworthy that metabolic rates and nutrient demands differ across species; therefore, the threshold effect of protein intake in humans may not be directly extrapolated from rodent models. The precise upper limit of safe daily protein intake in humans thus remains to be established through long-term, large-scale clinical investigations.

While short-term HP diet intake promotes weight loss and improves obesity-related metabolic disorders, long-term consumption has been linked to increased renal burden and elevated cardiovascular risk [4,6,57]. In our study, mice fed a long-term HP diet exhibited reduced adiposity without improved glucose tolerance, highlighting a disconnect between adiposity and systemic metabolic benefit. This uncoupling warrants caution against the use of body weight alone as a surrogate for dietary benefit, echoing the “lean-but-aging” phenotype observed in human populations consuming HP diets [6,58,59]. Mechanistically, we show that prolonged HP feeding induces adipocyte senescence. The accumulation of senescent adipocytes facilitates immune cell infiltration, particularly of pro-inflammatory macrophages, which in turn exacerbate adipose dysfunction through increased lipolysis, altered adipokine secretion, and impaired glucose handling [60,61]. Consistent with this, HP diet intake reduced adipocyte abundance while expanding immune cell populations (most prominently macrophages), concurrently with heightened inflammation and impaired insulin sensitivity. These findings align with well-established roles of the SASP in driving chronic inflammation and insulin resistance.

NAD^+^ homeostasis is closely associated with cellular senescence, with declining NAD^+^ levels have been observed in aging tissues across rodents and humans [26,62,63]. Our data reveal that in WAT, HP-induced NAD^+^ depletion in adipocytes is not driven by intrinsic metabolic reprogramming but rather by macrophage-derived CD38. This finding underscores the compartmentalized regulation of NAD^+^ metabolism during tissue aging. The concomitant alterations in TCA and pentose phosphate pathway intermediates further suggest that CD38 may impair mitochondrial function by restricting NAD^+^ availability, thereby influencing a broad spectrum of both NAD^+^-dependent and NAD^+^-independent processes, including redox homeostasis, cellular signaling, and epigenetic regulation. Immune cells can regulate NAD^+^ metabolism in adjacent parenchymal cells by modulating the availability of precursors [36]. In our study, genetic ablation of macrophage CD38 preserved adipocyte NAD^+^ pools, reduced senescence burden, and improved glucose tolerance, directly linking macrophage NADase activity to the acceleration of adipocyte senescence. These findings extend recent reports of macrophage NADase activity as a paracrine driver of inflammation, and underscore intercellular NAD^+^ exchange as a critical axis of tissue homeostasis.

Importantly, we demonstrate that NAD^+^ repletion*,* either through precursor supplementation or pharmacological CD38 inhibition, reverses HP-induced adipocytes senescence, even under sustained protein excess. This suggests that diet-induced metabolic aging is not irreversible. The restorative effect of NMN and 78c further emphasize the importance of NAD^+^ in maintaining adipocyte function. While previous NAD^+^ restoration strategies have primarily targeted biosynthetic pathways [64,65], our findings demonstrate that co-targeting degradation (e.g., CD38 inhibition) yields comparable benefits, thereby expanding the therapeutic landscape for age- and obesity-associated metabolic disorders. It is worth noting that peripheral tissues exhibit distinct patterns of bioavailability and responsiveness to NAD^+^ precursor supplementation, and their sensitivity to NAD^+^ depletion may differ substantially [26,[66], [67], [68], [69]]. In both mice and humans, skeletal muscle NAD^+^ levels decline by ∼10–30% with advancing age; however, genetic disruption of NAD^+^ synthesis in adult mice does not necessarily accelerate skeletal muscle aging [70]. In our study, we observed that NAD^+^ depletion promotes adipocyte senescence, which underscores the necessity of employing tissue-specific experimental models to elucidate the diverse roles of NAD^+^ metabolism across different organs during the aging process.

In summary, our study establishes that dietary protein content modulates adipocyte NAD^+^ metabolism through macrophage CD38 activity, thereby shaping the trajectory of adipocyte senescence. These findings position adipocyte NAD^+^ homeostasis as a key regulator of WAT aging and identify macrophage CD38 as a tractable therapeutic target for mitigating long-term diet-induced metabolic dysfunction.

## 4 Materials and Methods

All experiments were performed in accordance with institutional ethical guidelines and were approved by the licensing committee of Sichuan Agricultural University.

### 4.1 Animals

Wild-type 8-week-old C57BL/6J male mice were purchased from Vital River Laboratory Animal Technology Co., Ltd. (Beijing, China). C57BL/6J background *Cd38*^*fl/fl*^ (CKOAIP221017rm1) and *Lyz2*-cre(C001003) mice were obtained from Cyagen. Lyz2-cre; *Cd38*^*fl/fl*^ mice were generated by crossing *Cd38*^*fl/fl*^ mice with Lyz2-Cre mice. Male mice at 8-weeks old were used for experiments, with age-matched littermates WT mice (Lyz2wt/wt; *Cd38*^*fl/fl*^) as control. Mice were housed at the Animal Facility of Sichuan Agricultural University at room temperature (20–22 °C) with 12/12 dark/light system.

### 4.2 Diets

Mice were provided *ad libitum* one of the 3 diets varying in protein and carbohydrate content. All diets were custom designed and manufactured in dry, pelleted form by Dossy Experimental Animals Co. LTD (Chengdu, China), including a moderate-protein (MP, Cat.#D2014028-N) diet deriving energy 59.26% from carbohydrate, 17.97% from lipid and 22.49% from protein, a low-protein (LP, Cat.#D2014028-L) diet deriving energy 73.15% from carbohydrate, 17.87% from lipid and 9.07% from protein, whereas a high-protein (HP, Cat.#D2014028-H) diet deriving energy 25.44% from carbohydrate, 18.05% from lipid and 56.12% from protein. The food intake and body weight were determined every week.

### 4.3 Glucose tolerance test

After overnight fasting, blood sample and glucose measure was obtained from a small cut in the tail vein immediately before the intraperitoneal injection of d-glucose (1 g/kg body weight). Blood glucose levels were then measured at 0, 15, 30, 45, 60, 75, 90, 105, and120 min post-injection. Blood samples were taken from tail vein and tested with glucose test strips.

### 4.4 Body composition analysis

Body composition of mice was measured at the end of the dietary intervention using a quantitative magnetic resonance (QMR) system according to the manufacturer's instructions. Mice were placed in a restrainer without anesthesia and scanned to determine fat mass, lean mass, and total body water. Each measurement was repeated at least twice per animal to ensure accuracy, and the average value was used for subsequent analysis.

### 4.5 Senescence-associated β-galactosidase staining

Tissues were excised and immediately washed three times with phosphate-buffered saline (PBS). The samples were then fixed in SA-β-galactosidase (SA-β-gal) staining fixative solution (Beyotime Biotechnology, C0602) for 15 min at room temperature. After fixation, the tissues were washed again three times with PBS and incubated with SA-β-gal staining solution (Beyotime Biotechnology, C0602) at 37 °C for 10 h.

### 4.6 Histology and immunostaining

All tissues were fixed in 4% paraformaldehyde for 24 h at room temperature, dehydrated and embedded into paraffin. For immunofluorescence staining, paraffin-embedded sections were deparaffinized, subjected to epitope retrieval in citrate buffer (pH 6.0) using an autoclave for 15 min, rinsed with 0.05% Triton X-100 in PBS, and blocked with 1% BSA in PBS containing 0.05% Tween-20. Sections were incubated overnight at 4 °C with primary antibodies against PLIN1 (ab3526; Abcam), P21 (ab188224; Abcam), and CD68 (ab283654; Abcam), followed by appropriate Alexa Fluor-conjugated secondary antibodies. Nuclei were counterstained with DAPI. Images shown are representative of at least six biological replicates.

### 4.7 Metabolite measurements

Carbohydrates and small molecule metabolites were extracted from tissues using a modified method of Bligh and Dyer's extraction containing appropriate concentrations of internal standards including 13C5-NAD, 13C6-UDP-Glucose, 13C6-Glucose-6-Phosphate, 13C6-Fructose-6-Phosphate, 13C3,15N-NAM, 13C6-Fructose-1,6-bisphoshate, 2,3,3-d3-malic acid, 2,2,3,3-d4-succinic acid, 13C,15N2-Urea, 2,2,4,4-d4-citric acid, and l-Lactate-d3. The aqueous phase was extracted and dried in SpeedVac under aqueous mode. Samples were resuspended in acetonitrile: water (1:1 v/v). Samples were analysed on a Shimadzu 40X3B-UPLC coupled to Sciex 6500 Plus QTRAP under the electrospray ionization mode. Small molecule metabolites were separated on a Atlantis Premier BEH Z-HILIC column (100 × 2.1 mm, 1.7 μm) using mobile phase A (15 mM ammonium bicarbonate in H2O, pH 9) and mobile phase B (15 mM ammonium bicarbonate in H2O: acetonitrile 1:9, pH 9). Individual metabolites were quantitated by referencing to the intensities of their corresponding deuterated internal standards.

### 4.8 Quantitative PCR (q-PCR) analysis

We extracted total RNA using TRIzol reagent (Thermo Fisher Scientific) and purified it using RNA mini columns (Takara Bio). We synthesized cDNA from 1 μg of RNA and performed q-PCR in a 10 μL reaction volume with SYBR Green. The primer sequences for the genes detected are listed in Table S1.

### 4.9 Cytosolic mt-DNA detection

Cytosolic fractions were isolated from epididymal white adipose tissue after homogenization using the NE-PER™ Nuclear and Cytoplasmic Extraction Reagents (78833; Thermo Fisher Scientific), followed by differential centrifugation, and DNA was extracted. Cytosolic mt-DNA copy number was quantified by qPCR using mitochondrial and nuclear gene primers.

### 4.10 Transmission electron microscopy of eWAT mitochondria

Fresh eWAT was cut into ∼1 mm^3^ pieces and immediately fixed in 2.5% glutaraldehyde (0.1 M cacodylate buffer, pH 7.4) at 4 °C. Samples were rinsed, post-fixed in 1% osmium tetroxide, dehydrated through a graded ethanol series, and embedded in epoxy resin. Ultrathin sections (∼70 nm) were cut, mounted on copper grids, stained with uranyl acetate and lead citrate, and examined under a transmission electron microscope operated at 80–120 kV.

### 4.11 Western blot analysis

Tissues were homogenized using a FastPrep-24™ 5G (MP Biomedicals) in cold RIPA buffer containing protease inhibitors (Sigma–Aldrich). Lysates were centrifuged at 12,000 rpm for 30 min at 4 °C, and total protein was subjected to SDS-PAGE. Proteins were electrotransferred onto PVDF membranes and probed with antibodies against P16, P21, P53, CD38, and β-ACTIN. Protein levels were normalized to β-ACTIN for each sample.

### 4.12 Drug treatment

In the LPS and doxorubicin (doxo) experiments, 3- to 4-month-old male mice received daily intraperitoneal (i.p.) injections of LPS (20 μg/kg), doxo (2.5 mg/kg), or vehicle (PBS) for three consecutive days. For the 78c intervention, LPS or doxo was freshly mixed with the CD38 inhibitor 78c (20 mg/kg) and co-administered via i.p. Injection following the same schedule. Mice were euthanized 24 h after the final injection for sample collection.

For macrophage depletion experiments, animals were divided into four groups: LP (no injection), LP + PL (control liposomes), HP + PL, and HP + CL (clodronate liposomes). Clodronate (CL) or control PBS liposomes (PL) were administered i.p. (200 μL per injection) twice weekly from week 20, and mice were sacrificed at week 21.

For the supplementation experiments, mice were fed a LP or HP diet for 20 weeks and then randomly assigned to four groups: LP + PBS, HP + PBS, HP + NMN, and HP+78c. Animals received daily i.p. Injections of PBS, NMN (400 mg/kg), or 78c (20 mg/kg) for 2 weeks. Mice were sacrificed at week 22.

### 4.13 Flow cytometry analysis of ROS and macrophages in adipose tissue

The MAs and SVCs were isolated from eWAT depot. The mice were anesthetized and the fat pads were removed and digested in 0.1% (w/v) collagenase type Ⅱ (Invitrogen) for 30 min at 37 °C using a gentle MACSTM C tube (Miltenyi Biotec Inc., San Diego, CA, cat# 120-005-331) with the adapted WAT dissociation program 37c-mr-ATDK-1. The digestion mixture was passed through a 100 mm cell strainer (BD Biosciences) and centrifuged at 400×*g* for 10 min at 4 °C. After centrifugation, two phases are obtained, an upper phase that corresponds to MAs, while SVCs remain in the pellet. For measured the mitochondrial ROS, the MitoSOX™ Mitochondrial Superoxide Indicators (Thermo Fisher Scientific, cat# M36006) were used. The SVC pellets were collected and washed twice, 1 × 10^5^ freshly isolated cells were triple stained with F4/80-PE (Abcam, ab105156, clone CI:A3-1, 1:50), cd206-Alexa Fluor 647 (Biolegend, 141712, clone C068C2, 1:100) and cd11cFITC (BD Biosciences, BD 557400, clone HL3, 1:100) on ice for 30 min in dark. After staining, we performed cell analysis with a BD Verse Cell Analyzer (BD Biosciences) and analyzed the data using FlowJo software version X.0.7 (Tree Star, Inc.).

### 4.14 Single-nucleus RNA sequencing and data analysis

#### 4.14.1 Library preparation

We harvested tissues from mouse epididymal adipose and washed them in pre-cooled PBSE (PBS buffer containing 2 mM EGTA). Nuclei were isolated using GEXSCOPE® Nucleus Separation Solution (Singleron Biotechnologies, Nanjing, China) according to the manufacturer's instructions. Isolated nuclei were resuspended in PBSE at a concentration of 106 nuclei per 400 μl, filtered through a 40 μm cell strainer, and counted using Trypan blue. Nuclei enriched in PBSE were stained with DAPI (1:1,000) (Thermo Fisher Scientific, D1306) to identify DAPI-positive singlets. Subsequently, we conducted 2 × 150 bp paired-end sequencing (PE150) on a BGI DNBSEQ T7 instrument following the vendor's recommended protocol.

#### 4.14.2 Pre-processing, clustering and annotation

We used Singleron CeleScope software (v-1.15.0) to align reads from single-nucleus samples to the GRCm39 genome, generating gene count matrices. A total of 52,816 nuclei across four libraries from four samples were profiled. We identified and discarded “doublet” nuclei using the R package scDblFinder (v-1.4.0) [71]. Nuclei that did not meet our quality criteria were excluded based on three metrics: (i) genes expressed with non-zero countsgreater than 600; (ii) UMIs greater than 950; (iii) mitochondrial gene reads comprising less than median mitochondrial reads plus five times of MAD of mitochondrial reads. Additionally, genes expressed in fewer than 20 nuclei were filtered out. Following rigorous quality control, 35,165 nuclei remained for subsequent bioinformatic analyses. We integrated data from the four libraries across two conditions using Seurat (V-5.1.0) [72]. Gene expression counts were normalized and scale expression levels using “SCTransform”, while also regressing out the effects of mitochondrial proportion and cell cycle variability for each library. To correct for batch effects across samples and experiments, we utilized the top 1500 highly variable genes for canonical correlation analysis (CCA) implemented in Seurat. This approach facilitated alignment of samples. We conducted clustering of total nuclei using the “FindClusters” function with a resolution of 0.5, employing the first 30 principal components to define nuclei identities. Dimensionality reduction was achieved using the “RunUMAP” function and visualized via Uniform Manifold Approximation and Projection (UMAP). Subsequently, we annotated clusters of nuclei using cell type-specific markers identified from previous tissue-focused studies. Marker genes for each cell type in our atlas were determined using the Wilcoxon rank-sum test through the “FindAllMarkers” function, considering only those with “avg_logFC” > 0.25 and “p_val_adj” < 0.05 as significant markers. Nuclei originating from epididymal tissue were discarded, leaving 34,907 nuclei for downstream analyses. Pseudobulk expression profiles of adipocytes were generated using “AggregateExpression”, and differential expression was assessed using DESeq2 with adjusted *P*-values and log_2_ fold changes stabilized using the lfcShrink function [73].

#### 4.14.3 Pseudotime and potency score analysis

We utilized R package Monocle3 (v-1.3.7) to reconstruct the developmental trajectory of adipocyte nuclei [74]. Dimensionality reduction placed single cells in a two-dimensional space and connected them to construct a trajectory in a semi-supervised manner. Visualization functions such as “plot_cells” were employed to depict cells along a unified pseudotime trajectory.

#### 4.14.4 Transcriptional noise analysis

We employed a method to analyze the impact of obesity on various cell types through transcriptional noise, building upon previous studies [75]. Transcriptional noise was quantified for each cell type by analyzing a minimum of 10 cells from both LP-diet and HP-diet conditions. To standardize for differences in total UMI counts, all nuclei were down sampled to ensure equal library sizes. Additionally, to account for variations in cell-type frequency, snRNA-seq data were down sampled to 300 nuclei per cell type in each group; cell types with fewer than 300 nuclei included all available nuclei. Next, genes were categorized into ten blocks based on their average expression levels, and genes with the lowest coefficient of variation (CV) in each block (the lowest 10% CV) were selected. The Euclidean distance between each nuclear was then calculated based on these genes, serving as a measure of transcriptional heterogeneity within each cell type.

#### 4.14.5 Cell type composition variation analysis

Differential abundance analysis was conducted using miloR (v1.10.0), a k-nearest neighbor graph-based framework [38]. To ensure balanced comparisons between any two groups, samples were down-sampled to equal cell numbers. A SingleCellExperiment object was created from the count matrix. Neighborhood graphs were constructed using “buildGraph” (k = 30, d = 50) and “makeNhoods” (prop = 0.2, k = 30, d = 50, refinement_scheme = ‘graph’).

Differential abundance was tested using “testNhoods”, with significant neighborhoods (spatial FDR ≤0.1) visualized via “plotDAbeeswarm” function. Changes in cell type composition were quantified by computing the local true sign rate (LTSR) [76], which estimates the probability that the direction of log_2_ fold change is correct given its mean and variance. A threshold of LTSR > 0.9 was used to define significantly altered cell types.

### 4.15 Metabolic flux analyses

The metabolic profiles of different cells were inferred using flux-based analysis modelling in COMPASS [51]. For this, we created an expression matrix for adipocytes and macrophages, these matrices were pooled then used to run COMPASS. Statistical analysis to compare conditions was performed with a Wilcoxon test for every reaction, using their COMPASS score. COMPASS plots consisted of both positive and negative reactions grouped by their defined subsystem.

### 4.16 Signature score definition

Diversity score: Highly variable genes (HVGs) for each annotated cell type were identified from the LP group and utilized as cell-type-specific gene sets. For each nucleus, a module score was calculated using the corresponding HVG gene set via the “AddModuleScore” function; these scores were then compared between LP and HP samples to derive the diversity score shown in Figure 2C.

To evaluate transcriptional changes associated with high-protein diet, we computed a SASP score using a predefined SASP gene set. Gene set activity scores were calculated with “AddModuleScore” function of Seurat. The inflammation response score (Fig. 2D) was calculated as a module score using the “AddModuleScore” function, based on the gene set annotated to the inflammation response pathway.

### 4.17 Statistics and reproducibility

The specific sample sizes (the *n* numbers) are provided in the figure legends and indicated by dots. All data were included in the analysis without any exclusions. Data collection and analysis were conducted without blinding to the experimental conditions. All statistical analyses were conducted using Prism, version 9 (GraphPad Software Inc.). All data are shown as mean ± s.e.m. Two-tailed unpaired Student's t-test was used for the comparison between two groups. One-way analysis of variance (ANOVA) or two-way ANOVA followed by Tukey's post hoc testin was used for the multiple comparisons. Values of *P* < 0.05 were considered statistically significant. Quantitative image analysis was conducted using ImageJ.

## CRediT authorship contribution statement

**Xiaohan Yang:** Writing – review & editing, Writing – original draft, Project administration, Data curation. **Lun Hua:** Writing – review & editing, Writing – original draft, Project administration, Investigation, Funding acquisition, Data curation, Conceptualization. **Dengfeng Gao:** Visualization, Software, Methodology, Formal analysis. **Yanni Wu:** Validation, Investigation, Formal analysis. **Yi Yang:** Methodology, Investigation, Formal analysis. **Xianyang Jin:** Visualization, Investigation, Formal analysis. **Xuemei Jiang:** Validation, Supervision, Resources. **Chao Jin:** Validation, Data curation. **Bin Feng:** Validation, Project administration. **Lianqiang Che:** Validation, Resources, Project administration, Funding acquisition, Data curation. **Shengyu Xu:** Writing – review & editing, Data curation. **Yan Lin:** Supervision, Resources, Project administration. **Long Jin:** Writing – review & editing, Software, Formal analysis. **Yong Zhuo:** Writing – review & editing, Validation, Data curation, Conceptualization. **Mingzhou Li:** Writing – review & editing, Writing – original draft, Software, Funding acquisition, Conceptualization. **De Wu:** Writing – review & editing, Supervision, Project administration, Funding acquisition, Conceptualization.

## Funding

This work was supported by National Key Research & Development Program of China (2022YFD1301200 to L.H.), the 10.13039/501100001809National Natural Science Foundation of China (32421005 and 32230102 to D.W., 32225046 and 32494802 to M.L., 32472948 to L.H.), the Science and Technology Projects of Xizang Autonomous Region of China (XZ202501ZY0147 to M.L.), and the earmarked fund for China Agricultural Research System (CARS-35 to L.C.).

## Declaration of competing interest

The authors declare no competing interests.

## Data Availability

Data will be made available on request.

## Footnotes

Appendix Supplementary data to this article can be found online at https://doi.org/10.1016/j.molmet.2025.102306.
