# Supplementary material for: Long-term high-protein diet intake accelerates adipocyte senescence through macrophage CD38-mediated NAD+ depletion
Source: Mol Metab. 2025 Dec 13;103:102306. doi: 10.1016/j.molmet.2025.102306 (PMC12808574; doi:10.1016/j.molmet.2025.102306)
Supplement: Multimedia component 1 [file mmc1.docx]

**SUPPLEMENTARY MATERIALS**


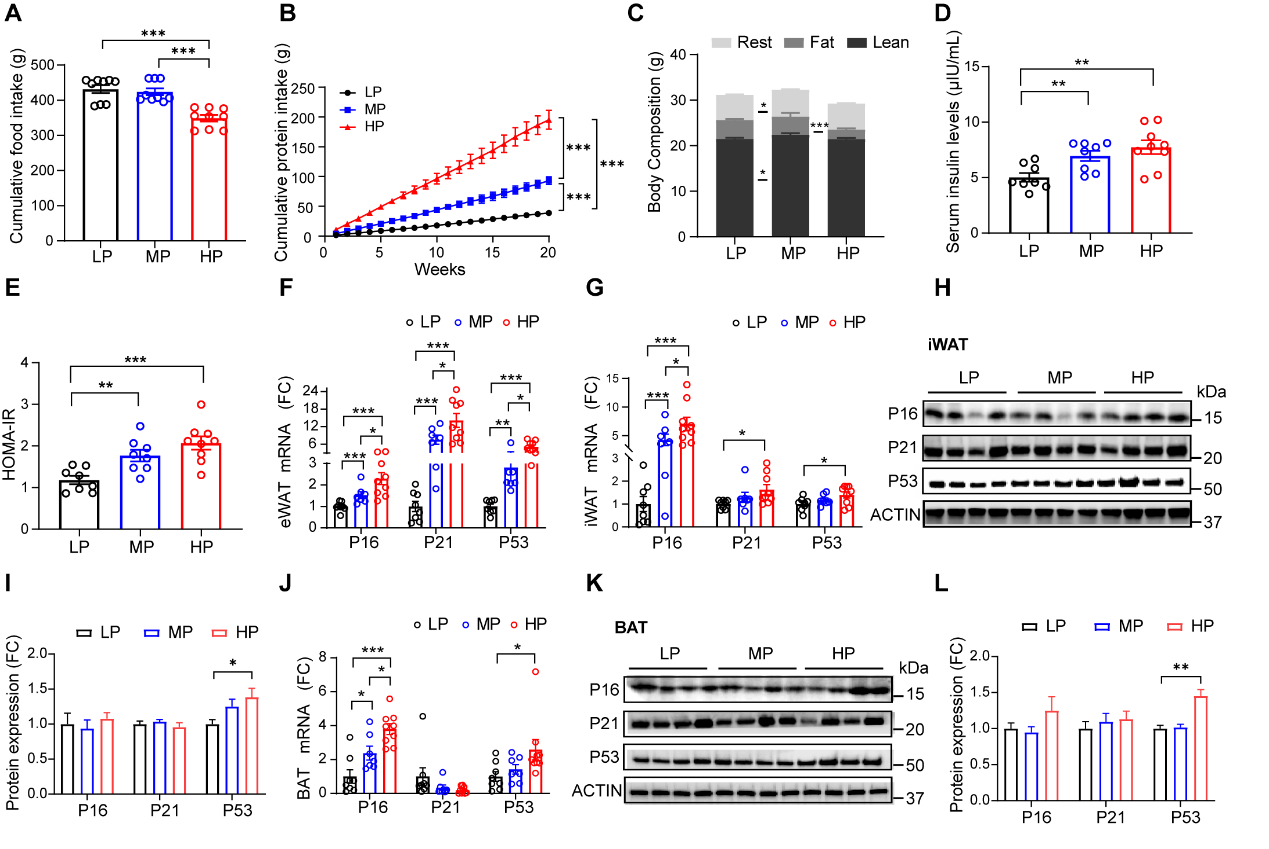


**Figure. S1. Long-Term HP Diets Induce Senescence in White Adipose Tissue but Not in Brown Adipose Tissue.**

(A) Cumulative food intake in 20-week (*n* = 9).

(B) Cumulative protein intake in 20-week (*n* = 9).

(C) Body composition measured by EchoMRI (*n* = 9).

(D,E) Serum insulin levels (D) and insulin resistance index (E) measured at week 18 (LP, MP, *n* = 8; HP, *n* = 9).

(F) mRNA levels of senescence markers (P16, P21 and P53) in eWAT (LP, *n* = 8; MP, *n* = 7; HP, *n* = 9).

(G-I) mRNA and protein levels of senescence markers (P16, P21 and P53) in iWAT (LP, *n* = 8; MP, *n* = 7; HP, *n* = 9).

(J-L) mRNA and protein levels of senescence markers (P16, P21 and P53) in BAT (LP, *n* = 8; MP, *n* = 7; HP, *n* = 9).


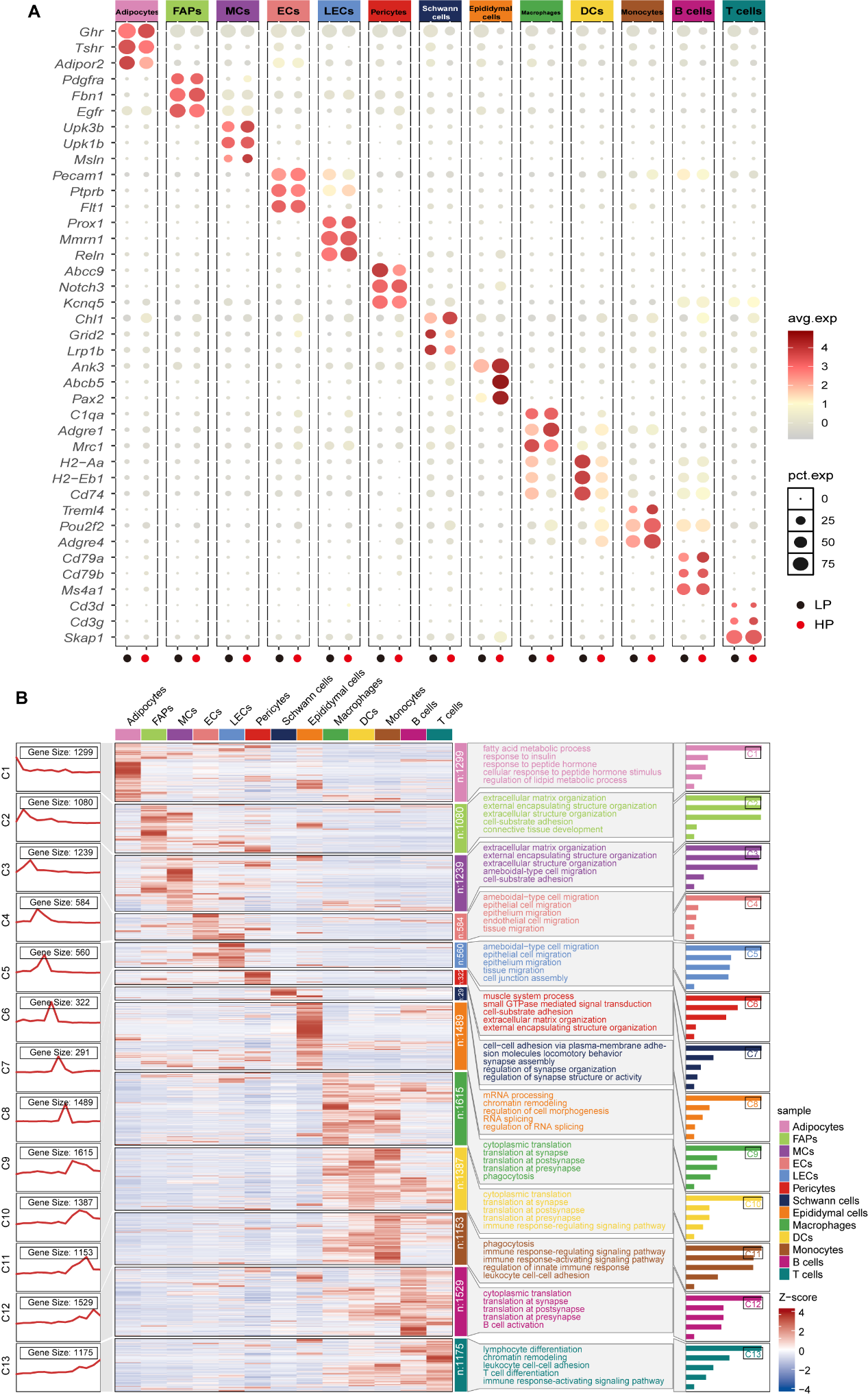


**Figure. S2. Additional snRNA-seq data.**

(A) Dot plot showing the expression of canonical marker genes for each annotated cell type in the snRNA-seq dataset from eWAT. Dot size corresponds to the fraction of cells expressing the gene, and color intensity indicates mean expression level.

(B) Enrichment terms of cell type-specific expressed genes. (Left) Line chart illustrating the expression changes of marker genes identified in different cell types. (Left-Middle) Heatmap depicting the expression levels of marker genes across various cell types. (Right-Middle) Top five significant GO-BP terms derived from the enrichment analysis of marker genes in different cell types. (Right) Bar chart showing the top five GO-BP terms, with -log_10_ (p-values) plotted along the X-axis.


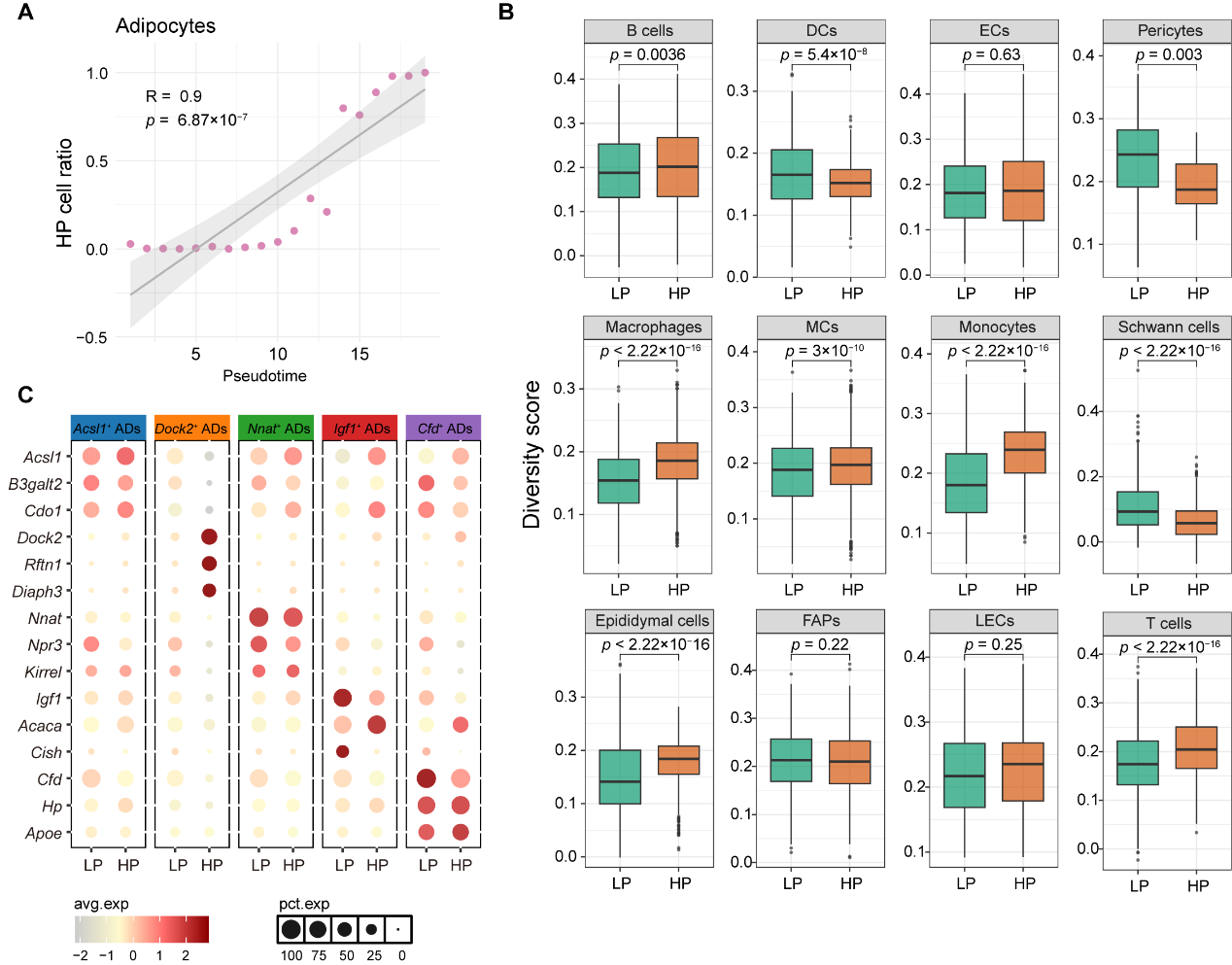


**Figure. S3. Additional snRNA-seq data of adipocytes.**

(A) Proportion of HP adipocyte nuclei along pseudotime.

(B) Box plot showing the diversity score of each cell type.

(C) Dot plot showing the expression of marker genes characteristic of each adipocyte subpopulation.

**Table S1. Forward and reverse primer sequences for target genes**

| Gene name |  | Sequence |
| --- | --- | --- |
| *mt-ND1* | F | TCACTATTCGGAGCTTTACGAGC |
|  | R | CATATTATGGCTATGGGTCAGGC |
| *18S rDNA* | F | TAGAGGGACAAGTGGCGTTC |
|  | R | CGCTGAGCCAGTCAGTGT |
| *Il1b* | F | CTGTGTCTTTCCCGTGGACC |
|  | R | CAGCTCATATGGGTCCGACA |
| *Tnf* | F | CAGCCTCTTCTCATTCCTGC |
|  | R | GGTCTGGGCCATAGAACTGA |
| *Il6* | F | ACAACCACGGCCTTCCCTACTT |
|  | R | CACGATTTCCCAGAGAACATGTG |
| *Ccl2* | F | CCACTCACCTGCTGCTACTCA |
|  | R | TGGTGATCCTCTTGTAGCTCTCC |
| *Adgre1* | F | CTTTGGCTATGGGCTTCCAGTC |
|  | R | GCAAGGAGGACAGAGTTTATCGTG |
| *p21* | F | CCCGCCTTTTTCTTCTTAGC |
|  | R | TTCTCATGCCATTCCTTTCC |
| *p16* | F | CCCAACGCCCCGAACT |
|  | R | GCAGAAGAGCTGCTACGTGAA |
| *p53* | F | AGAGACCGCCGTACAGAAGA |
|  | R | CTGTAGCATGGGCATCCTTT |
| *β-Actin* | F | GGCTGTATTCCCCTCCATCG |
|  | R | CCAGTTGGTAACAATGCCATGT |
